# Automatic Modulation Classification Based on Deep Learning for Unmanned Aerial Vehicles

**DOI:** 10.3390/s18030924

**Published:** 2018-03-20

**Authors:** Duona Zhang, Wenrui Ding, Baochang Zhang, Chunyu Xie, Hongguang Li, Chunhui Liu, Jungong Han

**Affiliations:** 1 School of Electronics and Information Engineering, Beihang University, Beijing 100083, China; zhangduona@buaa.edu.cn; 2 Unmanned Systems Research Institute, Beihang University, Beijing 100083, China; ding@buaa.edu.cn (W.D.); liuchunhui2134@buaa.edu.cn (C.L.); 3 School of Automation Science and Electrical Engineering, Beihang University, Beijing 100083, China; bczhang@buaa.edu.cn (B.Z.); yuxie@buaa.edu.cn (C.X.); 4 School of Computing & Communications, Lancaster University, Lancaster LA1 4WA, UK; jungonghan77@gmail.com

**Keywords:** deep learning, automatic modulation classification, classifier fusion, convolutional neural network, long short-term memory

## Abstract

Deep learning has recently attracted much attention due to its excellent performance in processing audio, image, and video data. However, few studies are devoted to the field of automatic modulation classification (AMC). It is one of the most well-known research topics in communication signal recognition and remains challenging for traditional methods due to complex disturbance from other sources. This paper proposes a heterogeneous deep model fusion (HDMF) method to solve the problem in a unified framework. The contributions include the following: (1) a convolutional neural network (CNN) and long short-term memory (LSTM) are combined by two different ways without prior knowledge involved; (2) a large database, including eleven types of single-carrier modulation signals with various noises as well as a fading channel, is collected with various signal-to-noise ratios (SNRs) based on a real geographical environment; and (3) experimental results demonstrate that HDMF is very capable of coping with the AMC problem, and achieves much better performance when compared with the independent network.

## 1. Introduction

Communication signal recognition is of great significance for several daily applications, such as operator regulation, communication anti-jamming, and user identification. One of the main objectives of signal recognition is to detect communication resources, ensuring safe, stable, timely, and reliable data exchange for communications. To achieve this objective, automatic modulation classification (AMC) is indispensable because it can help users identify the modulation mode within operating bands, which benefits communication reconfiguration and electromagnetic environment analysis. Besides this, AMC plays an essential role in obtaining digital baseband information from the signal when only limited knowledge about the parameters is available. Such a technique is widely used in both military and civilian applications, e.g., intelligent cognitive radio and anomaly detection, which have attracted much attention from researchers in the past decades [1,2,3,4,5,6].

Existing AMC algorithms can be divided into two main categories [3], namely, likelihood-based (LB) methods and feature-based (FB) methods. LB methods require calculating the likelihood function of received signals for all modulation modes and then making decisions in accordance with the maximum likelihood ratio test [3]. Even though LB methods usually obtain high accuracy and minimize the probability of mistakes, such methods suffer from high-latency classification or require complete priori knowledge, e.g., clock frequency offset. Alternatively, a traditional FB method consists of two parts, namely, feature extraction and classifier, where the classifier identifies digital modulation modes in accordance with the effective feature vectors extracted from the signals. Unlike the LB methods, the FB methods are computationally light but may not be theoretically optimal. To date, several FB methods have been validated as effective for the AMC problem. For instance, they successfully extract features from various time domain waveforms, such as cyclic spectrum [4], high-order cumulant [6], and wavelet coefficients. Afterwards, a classifier is used for final classification based on features mentioned above. With the development of learning algorithms, performances have been improved, such as with the shallow neural network [7] and decision tree for the support vector machine (SVM). Recently, deep learning has been widely applied to audio, image, and video processing, facilitating applications such as facial recognition and voice discrimination [8]. However, few works have been done based on deep learning in the field of communication.

Although researchers have developed various algorithms to implement AMC of digital signals, there are no representative data sets in the field of communication. Meanwhile, these methods are suitable for complex communication equipment and struggle in real-world applications where channels are variable and difficult to predict, because (1) their samples are purely theoretical without the information of real geographical environment; (2) they usually separate feature extraction and the classification process so that information loss is inevitable; and (3) they employ handcrafted features which contribute to the lack of characterization capabilities. In this paper, we propose to realize AMC using convolutional neural networks (CNNs) [9], long short-term memory (LSTM) [10], and a fusion model to directly process the time domain waveform data, which is collected with various signal-to-noise ratios (SNRs) based on a real geographical environment.

CNNs exploit spatially local correlation by enforcing a local connectivity pattern between neurons of adjacent layers. The convolution kernels are also shared in each sample for the rapid expansion of parameters caused by the fully connected structure. Sample data are still retained in the original position after convolution such that the local features are well preserved. Despite its great advances in spatial feature extraction, CNNs cannot model the changes in time series well. As is known to us, the temporal property of data is important for AMC applications. As a variant of the recurrent neural network (RNN), LSTM uses the gate structure to realize information transfer in the network in time sequence, which reflects the depth in time series. Therefore, LSTM has a superior capacity to process the time series data.

**Figure 1:**
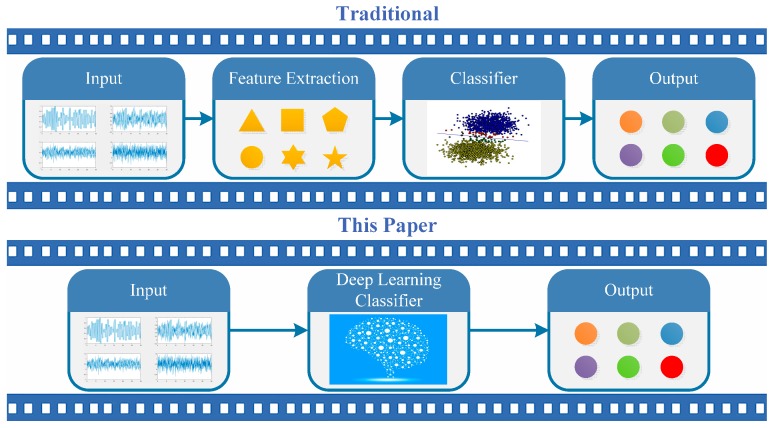
Illustration of the traditional and classifier methods in this study for automatic modulation classification (AMC). The traditional methods usually separate feature extraction and the classification process. Meanwhile, they usually employ handcrafted features, which might contribute to limitations in representing the samples. By contrast, we deploy deep learning to solve the AMC problem, due to its high capacity for feature representation. In addition, deep learning is generally performed in the end-to-end framework, which performs the feature extraction and classification in the same process. Our deep methods achieve a much lower computational complexity during testing compared with the training process. The upshot is that AMC is implemented more efficiently with a heterogeneous deep model fusion (HDMF) method.

This paper proposes a heterogeneous deep model fusion (HDMF) method to solve the AMC problem in a unified framework. The framework is shown in Figure 1. Different from using conventional methods, we solve feature extraction and classification in a unified framework, i.e., based on end-to-end deep learning. In addition, high-performing filters can be obtained based on a learning mechanism. This improvement helps the communication system achieve a much lower computational complexity during testing when compared with the training process. As a further result, an accurate classification performance can be achieved due to its high capacity for feature representation. We use CNNs and LSTM to process the time domain waveforms of the modulation signal. Eleven types of single-carrier modulation signal samples (e.g., MASK, MFSK, MPSK, and MQAM) with additive white Gaussian noise (AWGN) and a fading channel are generated under various signal-to-noise ratios (SNRs) based on an actual geographical environment. Two kinds of HDMFs based on the serial and parallel modes are proposed to increase the classification accuracy. The results show that HDMFs achieve much better results than the single CNN or LSTM method, when the SNR is in the range of 0–20 dB. In summary, the contributions are as follows:

(1) CNNs and LSTM are fused based on the serial and parallel modes to solve the AMC problem, thereby leading to two HDMFs. Both are trained in the end-to-end framework, which can learn features and make classifications in a unified framework.
(2) The experimental results show that the performance of the fusion model is significantly improved compared with the independent network and also with traditional wavelet/SVM models. The serial version of HDMF achieves much better performance than the parallel version.
(3) We collect communication signal data sets which approximate the transmitted wireless channel in an actual geographical environment. Such datasets are very useful for training networks like CNNs and LSTM.

The rest of this paper is organized as follows: Section 2 briefly introduces related works. Section 3 introduces the principle of the digital modulation signal and deep learning classification methods. Section 4 presents the experiments and analysis. Section 5 summarizes the paper.

## 2. Related Works

AMC is a typical multiclassification problem in the field of communication. This section briefly introduces several feature extraction and classification methods in the traditional AMC system. The CNN and LSTM models are also presented.

### 2.1. Conventional Works Based on Separated Features and Classifiers

Traditionally the features and classifier are separately built for an AMC system. For example, the envelope amplitude of signal, the power spectral variance of signal, and the mean of absolute value signal frequency were extracted in [11] to describe a signal from several different aspects. Yang and Soliman used the phase probability density function for AMC [12]. Meanwhile, traditional methods usually combine instantaneous and statistical features. Shermeh used the fusion of high-order moments and cumulants with instantaneous features for AMC [13,14]. The features can describe the signals using both absolute and relative levels. In addition, the high-order features can eliminate the effects of noise. The eighth statistics are widely used in several methods.

Classical algorithms have been widely used in the AMC system. Panagiotou et al. considered AMC as a multiple-hypothesis test problem and used decision theory to obtain the results [15]. They assumed that the phase of AWGN was random and dealt with the signals as random variables with known probability distribution. Finally, the generalized likelihood ratio test or the average likelihood ratio test was used to obtain the classification results by the threshold. The classifiers were then used in the AMC system. In [16], shallow neural networks and SVM were used as classifiers. In [17,18], modulation modes were classified using CNNs with high-level abstract learning capabilities. However, the traditional classifiers are let down either by their capacity for feature representation or by requiring complete priori knowledge, e.g., clock frequency offset. This approach has led to negative influences on the classification performance.

Recently, accompanied with a probabilistic-based output layer, sparse autoencoders based on deep neural networks (DNNs) were introduced for AMC [19,20]. These methods showed the promising potential of the deep learning model for the AMC task. Instead, we propose heterogeneous deep model fusion (HDMF) methods which combine CNN and LSTM to learn the spatially local correlations and temporal properties of communication signals based on an end-to-end framework. The main difference from previous works [19,20] lies in the exploitation of different kinds of features in the combinations of CNN and LSTM. The HDMFs are capable of obtaining high-performing filters based on a learning mechanism, and achieve a much lower computational complexity level during testing.

### 2.2. CNN-Based Methods

The advantage of CNNs is achieved with local connections and tied weights followed by some form of pooling which results in translation-invariant features. Furthermore, another benefit is that they have many fewer parameters than do fully connected networks with the same number of hidden units. In [9], the authors treated the communication signal as 2-dimensional data, similar to an image, and took it as a matrix to a narrow 2D CNN for AMC. They also studied the adaptation of CNN to the time domain in-phase and quadrature (IQ) data. A 3D CNN was used in [21,22] to process video information. The result showed that CNN multiframes were considerably more suitable than a single-frame network for video cognition. In [23], Luan et al. proposed Gabor Convolutional Networks, which combine Gabor filters and a CNN model, to enhance the resistance of deep-learned features to orientation and scale changes. Recently, Zhang et al. applied a one-two-one network to compression artifact reduction in remote sensing [24]. This motivates us to solve the AMC problem.

### 2.3. LSTM-Based Methods

Various models have been used to process sequential signals, such as hidden semi-Markov models [25], conditional random fields [26], and finite-state machines [27]. Recently, RNN has become well known with the development of deep learning. As a special RNN, LSTM has been widely used in the field of voice and video because of its ability to handle gradient disappearance in traditional RNNs. It has fewer conditional independence hypotheses compared with the previous models and facilitates integration with other deep learning networks. Researchers have recently combined spatial/optical flow CNN features with vanilla LSTM models for global temporal modeling of videos [28,29,30,31,32]. These studies have demonstrated that deep learning models have a significant effect on action recognition [29,31,33,34,35] and video description [32,36,37]. However, to our best of knowledge, the serial and parallel fusion of CNN and LSTM has never before been investigated to solve the AMC problem at the same time.

## 3. Heterogeneous Deep Model Fusion

### 3.1. Communication Signal Description

The samples in this paper were collected via a realistic process with due consideration for the communication principle and real geographical environment. The received signal in the communication system can be expressed as follows:

(1) y(t)=x(t)⋅c(t)+n(t)

where x(t) is the efficient signal from the transmitter, c(t) represents the transmitted wireless channel on the basis of the actual geographical environment, and n(t) denotes the AWGN. The communication signal in general is divided into three parts to start with.

#### 3.1.1. Modulation Signal Description

The digital modulation signal x(t) from the transmitter can be expressed as follows:

(2) $$x(t)=(A_{c}+jA_{s})e^{j(2\pi ft+\theta )}g(t-nT)=(A_{c}\cos(2\pi ft+\theta )-A_{s}\sin(2\pi ft+\theta ))g(t-nT),0\leq t\leq NT$$

where $A_{c}$ and $A_{s}$ are the amplitudes of the in-phase and quadrature channel, respectively; f stands for the carrier frequency; θ is the initial phase of the carrier; and g(t−nT) represents the digital sampling pulse signal. In the case of ASK, FSK, and PSK, $A_{s}$ is zero. In accordance with the digital baseband information, ASK, FSK, and PSK change $A_{c}$, f, and θ in the range of 0−M, 1−M, and 0−2π/M, respectively, over time. By contrast, QAM fully utilizes the orthogonality of the signal. After dividing the digital baseband into I and Q channels, the information is integrated into two identical frequency carriers with phase difference of 90° using the ASK modulation mode, which significantly improves the bandwidth efficiency.

The sampling rate of data is 20 times as much the carrier frequency and 60 times as much as the symbol rate; in other words, a symbol period contains three complete carrier waveforms and a carrier period is made of 20 sample dots. Meanwhile, the carrier frequency scope is broadband, in the frequency range of 20 MHz to 2 GHz.

#### 3.1.2. Radio Channel Description

The Longley-Rice model (LR) is an irregular terrain model for radio propagation. We use this method for predicting the attenuation of communication signals for a point-to-point link. LR is proposed for different scenarios and heights of channel antennas in the frequency range of 20 MHz to 20 GHz. This model applies statistics to modify the characterization of the channel, which depends on the variables of each scenario and environment. It determines variation in the signal by the prediction method based on atmospheric changes, topographic profile, and free space. The variations are deformed under actual situation information, such as permittivity, polarization direction, refractive index, weather pattern, and so on, which have deviations that contribute to the attenuation of the signal. The attenuation can be roughly divided into three kinds according to transmission distance as follows:

(3) $$A_{ref}(d)=\{\begin{array}{c} A_{el}+k_{1}d+k_{2}\log d,\\ A_{ed}+m_{d}d,\\ A_{es}+m_{d}d, \end{array}\begin{array}{c} d_{\min}<d<d_{Ls}\\ d_{Ls}<d<d_{x}\\ d>d_{x} \end{array}$$

where $d_{\min}<d<d_{Ls}$, $d_{Ls}<d<d_{x}$, and $d>d_{x}$ represent the transmission distances in the range of line-of-sight, diffraction, and scatter, respectively. The value of d is determined by the real geographic coordinates of communication users.

As one of the most common types of noise, AWGN is always true whether or not the signal is in the communication system. The power spectrum density is a constant at all frequencies, and the noise amplitude obeys the Gauss distribution.

### 3.2. CNNs

CNNs are a hierarchical neural network type that contain convolution, activation, and pooling layers. In this study, the input of the CNN model is the data of the signal time domain waveform. The difference among the classes of modulation methods is deeply characterized by the stacking of multiple convolutional layers and nonlinear activation. Different from the CNN models in the image domain, we use a series of one-dimensional convolution kernels to process the signals.

Each convolution layer is composed of a number of kernels with the same size. The convolution kernel is common to each sample; thus, each kernel can be called a feature extraction unit. This method of sharing parameters can effectively reduce the number of learning parameters. Moreover, the feature extracted from convolution remains in the original signal position, which preserves the temporal relationship well within the signal. In this paper, rectified linear unit (ReLU) is used as the activation function. We do not use the pooling layer for dimensionality reduction because the amount of signal information is relatively small.

### 3.3. LSTM

**Figure 2:**
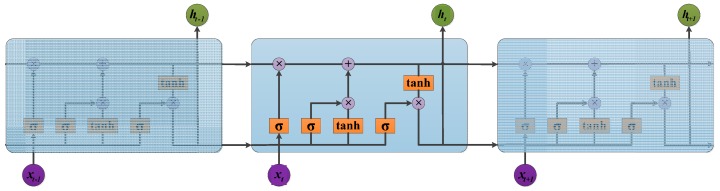
Long short-term memory (LSTM) memory cell structure.

Traditional RNNs are unable to connect information as the gap grows. The vanishing gradient can be interpreted as like the process of forgetting in the human brain. LSTM overcomes this drawback using gate structures that optimize the information transfer among memory cells. The particular structures in memory cells include the input, output, and forget gates. An LSTM memory cell is shown in Figure 2.

The iterating equations are as follows:

(4) $$f_{t}=sig\mathrm{mod}(W_{f}\cdot [h_{t-1},x_{t}]+b_{f})$$

(5) $$i_{t}=sig\mathrm{mod}(W_{i}\cdot [h_{t-1},x_{t}]+b_{i})$$

(6) $${\overset{\wedge }{C}}_{t}=\tanh(W_{C}\cdot [h_{t-1},x_{t}]+b_{C})$$

(7) $$C_{t}=f_{t}\cdot C_{t-1}+i_{t}\cdot {\overset{\wedge }{C}}_{t}$$

(8) $$o_{t}=sig\mathrm{mod}(W_{o}\cdot [h_{t-1},x_{t}]+b_{o})$$

(9) $$h_{t}=o_{t}\cdot \tanh(C_{t})$$

where W is the weight matrix; b is the bias vector; i, f, and o are the outputs of the input, forget, and output gates, respectively; C and h are the cell activations and cell output vectors, respectively; and sigmod and tanh are nonlinear activation functions.

Standard LSTM usually models the temporal data in the backward direction but ignores the forward temporal data, which has a positive impact on the results. In this paper, a method based on bidirectional LSTM (Bi-LSTM) is exploited to realize AMC. The core concept is to use a forward and a backward LSTM to train a sample simultaneously. Similarly, the architecture of the Bi-LSTM network is designed to model time domain waveforms from past and future.

### 3.4. Fusion Model Based on CNN and LSTM

**Figure 3:**
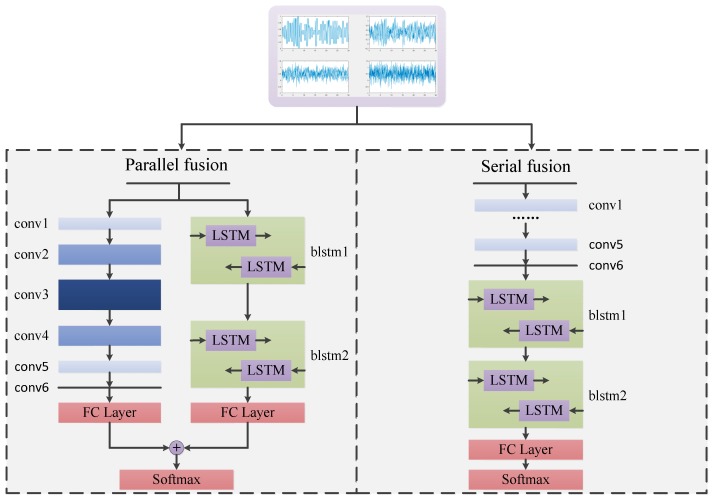
Fusion model structure of heterogeneous deep model fusion (HDMF) in parallel and series modes. We note that two HDMF models are used separately to solve the AMC problem.

The HDMFs are established based on the fusion model in serial and parallel ways to enhance the classification performance. The specific structure of the fusion model is shown in Figure 3.

The modulated communication signal has local special change features. Meanwhile, the data has temporal features similar to voice and video. The fusion models exploit complementary advantages on the basis of these two features.

The six layers of CNNs are used to characterize the differences between the digital modulation modes in the fusion model. The kernel numbers of the convolutional layers are different for each layer. The number of convolutional kernels in the first three layers increases gradually, which transforms single-channel into multichannel signal data. Such a transformation also helps to obtain effective features. Conversely, the number of convolutional kernels in the remaining layers reduces gradually. Finally, the result is restored to single-channel data. Although the data format is the same as the original signal, local features of the signal are extracted by multiple convolution kernels. This leads to the representation for the final classification based on CNNs. The remaining part of the fusion model uses the two-layer Bi-LSTM network to learn the temporal correlation of signals. The output of the upper Bi-LSTM is used as the input for the next layer.

**The parallel fusion model (HDMF).** The two networks are used to train samples simultaneously. The output of each network is then transformed into an 11-dimensional feature vector by the full connection layer. The resulting feature vectors represent the judgment of the modulation modes of the training samples by the two networks. We then combine the two vectors based on the sum operation as:

(10) $$\ell_{total}=\omega_{c}\cdot \ell_{c}+\omega_{l}\cdot \ell_{l}$$

and

(11) $$\omega_{c}+\omega_{l}=1,0\leq \omega \leq 1.$$

The loss function of the parallel fusion model consists of two parts, which are balanced by the given parameters.

In Algorithm 1, we show the optimization of the parallel fusion model.

**The serial fusion method (HDMF).** This is similar to the encoder–decoder framework. In this study, the encoding process is implemented by CNNs; afterwards, LSTM decodes the corresponding information. The features are extracted by the two networks, from simple representation to complex concepts. The upper convolutional layers can extract features locally. Then, the Bi-LSTM layers learn temporal features from these representations.

For both kinds of fusion models, the final feature vectors are the probabilistic output of the softmax layer. The fusion models are trained in the end-to-end way even when different neural networks are used to address the AMC problem.

**Algorithm 1.** Training HDMF (parallel)1: Initialize the parameters $\theta_{c}$ in CNN, $\theta_{l}$ in LSTM, W, ω in the loss layer, the learning rate μ, and the number of iterations t=0.2: **While** the loss does not converge, **do**3: t=t+14: Compute the total loss by $\ell_{total}=\omega_{c}\cdot \ell_{c}+\omega_{l}\cdot \ell_{l}$.5: Compute the backpropagation error $\frac{\partial \ell_{total}}{\partial x_{i}}$ for each $x_{i}$ by $\frac{\partial \ell_{total}}{\partial x_{i}}=\omega_{c}\cdot \frac{\partial \ell_{c}}{\partial x_{i}}+\omega_{l}\cdot \frac{\partial \ell_{l}}{\partial x_{i}}$.6: Update parameter W by $W-\mu \cdot \frac{\partial \ell_{total}}{\partial W}=W-\mu \cdot \omega_{c}\cdot \frac{\partial \ell_{c}}{\partial W}-\mu \cdot \omega_{l}\cdot \frac{\partial \ell_{l}}{\partial W}$7: Update parameters $\omega_{c}$ and $\omega_{l}$ by $\omega_{c,l}-\mu \cdot \frac{\partial \ell_{c,l}}{\partial \omega_{c,l}}$.8: Update parameter θ by $\theta_{c,l}-\mu \cdot \sum_{i}^{m}\frac{\partial \ell_{c,l}}{\partial x_{i}}\cdot \frac{\partial x_{i}}{\partial \theta_{c,l}}$.9: **End while**

### 3.5. Communication Signal Generation and Backpropagation

**Figure 4:**
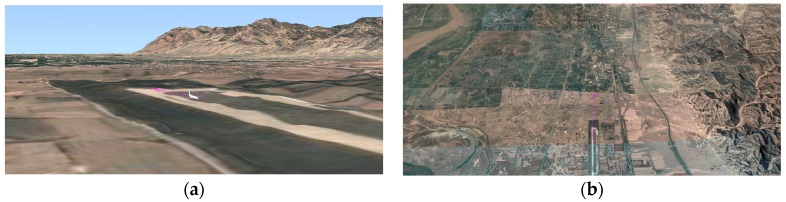
The geographic simulation environment. (**a**) Short-distance perspective of the real geographical environment; (**b**) Long-distance perspective of the real geographical environment.

The geographic simulation environment is shown in Figure 4; it was based on this environment that we collected our datasets. We captured the unmanned aerial vehicle communication signal dataset, which was developed by us based on Visual Studio, and MATLAB. These functions were integrated into a unified format. In Algorithm 2, we show the process of communication signal generation.

Detailed descriptions of the datasets are shown in Table 1.

**Algorithm 2.** Communication signal generation1: Open the real geographic environment through the control in Visual Studio.2: Real-time track transmission and simulation of unmanned aerial vehicle (UAV) flight.3: Add the latitude and longitude coordinates of the radiation and the height of the antenna.4: Build an LR channel model based on the parameters of coordinate, climate, and terrain, etc.5: Generation of baseband signals randomly and in order to generate various modulation signals by MATLAB.6: The communication between Visual Studio and MATLAB is by means of a User Datagram Protocol (UDP), and the real sample data is generated and finally stored.

We used TensorFlow [38] to implement our deep learning models. The experiments were done on a PC with an Nvidia GTX TITAN X GPU graphics card (Nvidia, Santa Clara, CA, USA), an Intel Core i7-6700K CPU (Nvidia, Santa Clara, CA, USA), and a 32 GB DDR4 SDRAM. The version of Cuda is 5.1. The Adam method [39] was used to solve our model with a 0.001 learning rate. The iterations are as follows:

(12) $$m_{t}=\mu \cdot m_{t-1}+(1-\mu )\cdot g_{t}$$

(13) $$n_{t}=\nu \cdot n_{t-1}+(1-\nu )\cdot g_{t}^{2}$$

(14) $$\overset{\wedge }{m_{t}}=\frac{m_{t}}{1-\mu^{t}}$$

(15) $$\overset{\wedge }{n_{t}}=\frac{n_{t}}{1-\nu^{t}}$$

(16) $$\Delta \theta =-\frac{\overset{\wedge }{m_{t}}}{\sqrt{\overset{\wedge }{n_{t}}}+\varepsilon }\cdot \eta$$

where $m_{t}$ and $n_{t}$ are the first and second moment estimations of the gradient, which represent the estimation of $E(g_{t})$ and $E(g_{t}^{2})$, respectively; $\overset{\wedge }{m_{t}}$ and $\overset{\wedge }{n_{t}}$ are the corrections of $m_{t}$ and $n_{t}$, respectively, which can be regarded as the unbiased estimation of expectation; Δθ is the dynamic constraint of learning rate; and μ, ν, ε, and η are constants.

The fundamental loss and the softmax functions are defined as follows:

(17) $$\ell (x,y)=-\log(p_{y})$$

(18) $$p_{y}=\frac{e^{z_{y}}}{\sum_{i}e^{z_{i}}}=\frac{e^{W_{y_{i}}^{T}x_{i}+b_{y_{i}}}}{\sum_{j=1}^{n}e^{W_{j}^{T}x_{i}+b_{j}}}$$

where x is the input, y is the corresponding truth label, and $z_{i}$ is the input for the softmax layer. The gradient of backpropagation [40] is calculated as follows:

(19) $$g_{t}=\frac{\partial \ell }{\partial z_{j}}=\frac{\partial \ell }{\partial p_{y}}\cdot \frac{\partial p_{y}}{\partial z_{j}}=-\frac{1}{p_{y}}p_{y}(I_{jy}-p_{j})=p_{j}-I_{jy}$$

where $I_{jy}=1$ if j=y, and $I_{jy}=0$ if j≠y.

## 4. Results

### 4.1. Classification Accuracy of CNN and LSTM Models

Using CNNs and LSTM to solve the AMC problem, the classification accuracies of CNNs are here reported for varying convolution layer depth from 1 to 4, number of convolution kernels from 8 to 64, and size of convolution kernels from 10 to 40. The classification accuracies of Bi-LSTM were tested with varying layer depth from 1 to 3 and number of memory cells from 16 to 128. The Bi-LSTM used in the fusion model contained two layers. The number of convolution layers was 6. The number of convolution kernels in the first three layers was 8, 16, and 32, and the size of the convolution kernel was 10. The number of convolution kernels in the remaining layers was 16, 8, and 1, and the size of the convolution kernel was 20. The Bi-LSTM model consisted of two layers with 128 memory cells.

**Figure 5:**
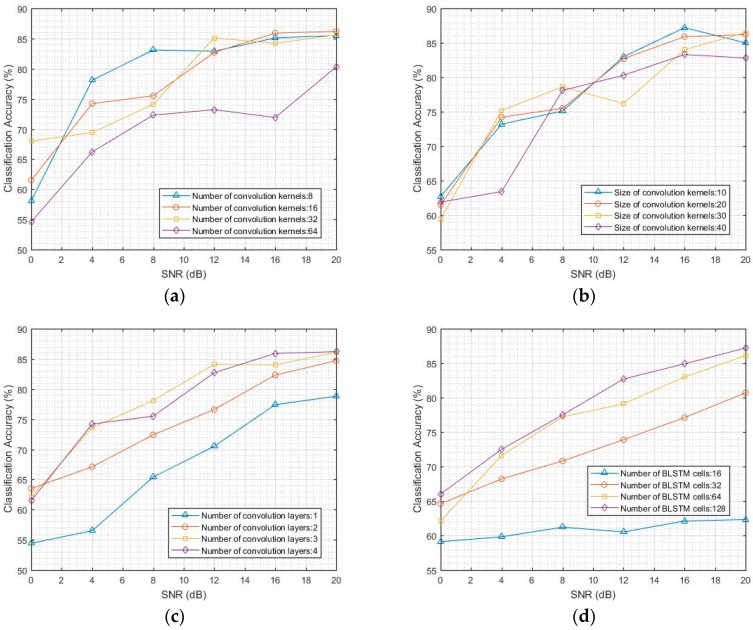

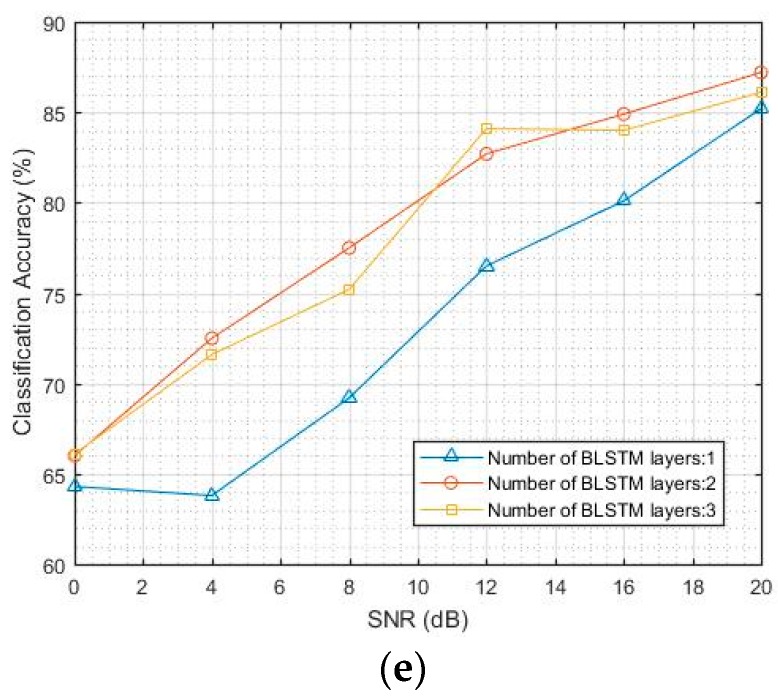
Classification accuracy of convolutional neural network (CNN) and LSTM models. (**a**) Classification accuracy of CNN when the number of convolution kernels is from 8 to 64; (**b**) Classification accuracy of CNN when the size of convolution kernels is from 10 to 40; (**c**) Classification accuracy of CNN when the number of convolution layers is from 1 to 4; (**d**) Classification accuracy of Bi-LSTM when the number of memory cells is from 16 to 128; (**e**) Classification accuracy of Bi-LSTM when the number of hidden layers is from 1 to 3.

For SNR from 0 dB to 20 dB, the classification accuracy of CNN and Bi-LSTM models is shown in Figure 5. The samples with SNR below 0 dB were not considered in this study. The classification results of the CNN models are shown in Figure 5a–c. The average classification accuracy of the CNN model for AMC can reach 75% for SNR from 0 dB to 20 dB. An excess of convolution kernels in each layer reduces the classification accuracy. The performance is better when the number of convolution kernels is from 8 to 32. The CNN models with convolution kernels of size 10 to 40 have more or less the same classification accuracy. Increasing the number of convolution layers from 1 to 3 results in a performance boost. The classification results of the Bi-LSTM models are shown in Figure 5d,e. The results show that the Bi-LSTM model is more suitable for AMC than the CNN model. The average classification accuracy of Bi-LSTM is 77.5%, which is 1.5% higher than that of the CNN model. The performance is better when the number of memory cells is from 32 to 128 than when the number is outside this range. The Bi-LSTM models with more than 2 hidden layers have essentially the same classification accuracy.

The training parameters and computational complexity of CNNs are shown in Table 2. The results reveal that the proportion of samples with training parameters is reasonable and that our CNNs achieve much lower computational complexity during testing.

### 4.2. Comparison of Classification Accuracy between the Deep Learning Models and the Traditional Method

We have compared five methods, including both traditional and deep learning methods, based on the same data sets. The classification performance is as follows.

**Figure 6:**
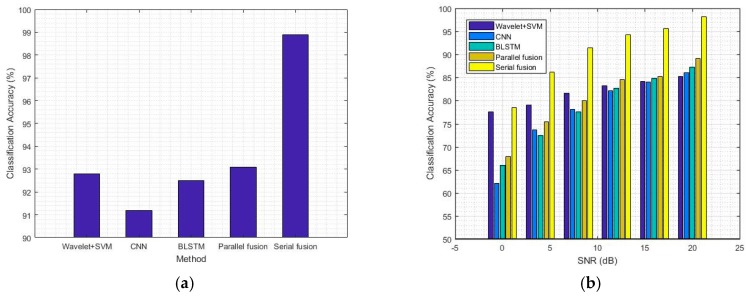
Comparison of classification accuracy between the deep learning models and the traditional method. (**a**) Classification accuracy of different methods without noise; (**b**) Classification accuracy of different methods with SNR from 0 dB to 20 dB.

The modified classifiers are established based on the fusion model in serial and parallel modes to increase the classification accuracy. As a result, we compare the classification accuracy of the methods on the basis of deep learning with the traditional method using wavelet and SVM classifiers. The results are shown in Table 3 and Table 4 and Figure 6. The results reveal that the fusion methods have a significant effect on improving classification accuracy. The average classification accuracy of the parallel fusion model is 93% without noise, which is equal to that of the traditional method. The classification accuracy of the parallel fusion model is 2% higher than that of the CNN model and 1% higher than that of the Bi-LSTM model. Moreover, the average classification accuracy of the serial fusion model is 99% without noise, which is 6% higher than that of the parallel fusion model. In fact, the fusion methods are more beneficial to the classification accuracy when the SNR is from 0 dB to 20 dB compared with in the noise-free situation. When the SNR is from 0 dB to 20 dB, the average classification accuracy of the serial fusion method is 91%, which is 11% higher than that of the parallel fusion method.

The performances of the classifiers show that deep learning achieves high classification accuracy for AMC. Waveform local variation and temporal features can be used to identify modulation modes. In comparison with CNN and Bi-LSTM, the performance of the HDMF methods is improved significantly because the classifiers can recognize the two features simultaneously. However, the performance of the serial fusion is considerably higher than that of the parallel fusion because the parallel method belongs to decision-level fusion. The fusion can be viewed as a simple voting process for results. The serial method belongs to feature-level fusion, which combines the feature information to obtain the classification results.

**Figure 7:**
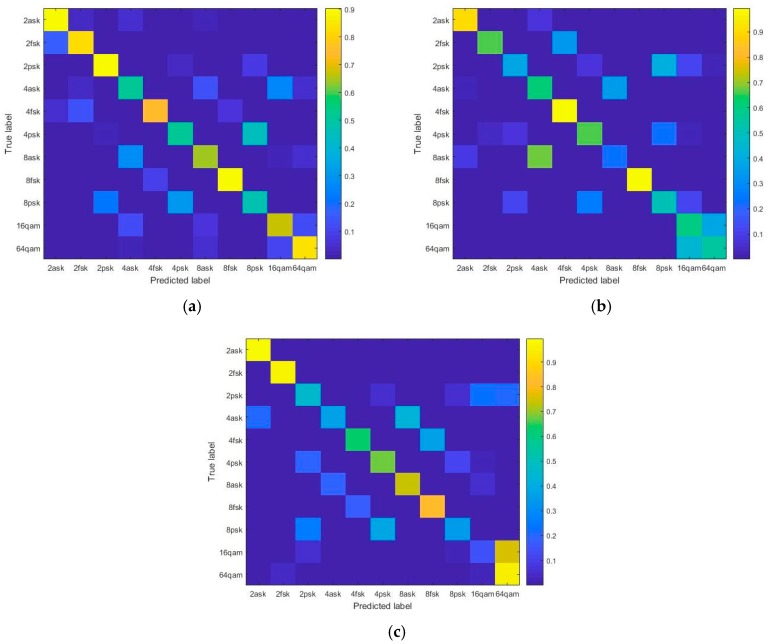
Probability matrix of series fusion model. (**a**) Probability matrix of series fusion model for 20 dB SNR; (**b**) Probability matrix of series fusion model for 10 dB SNR; (**c**) Probability matrix of series fusion model for 0 dB SNR.

In this study, the modulation mode of the samples includes two forms, namely, within-class and between-class modes. The probability matrices show the identification results of the modulation modes by the serial fusion model when the SNR is 20, 10, and 0 dB, respectively; the results are shown in Figure 7. When the SNR is 20 dB, a profound discrepancy is observed between the different modulation modes. The probability result does not have the error. The decrease of SNR, PSK, and QAM is prone to misclassification within class, caused by the subtle differences in the M-ary phase mode. Since the waveform variances of the carrier phase appear only once in each symbol period, such change is difficult to obtain in real time. Moreover, the waveform variances caused by phase offset might be neglected, attenuating and interfering under some circumstances. By contrast, the variances of amplitude and frequency are relatively stable. Furthermore, QAM can be considered as a combination of ASK and PSK in practice, which means that the waveforms have the amplitude and phase variances simultaneously. The classifier can detect the different types of variances even when the result is incorrect at low SNR. Therefore, only within-class misclassifications occur in the results.

## 5. Conclusions

In this study, we proposed methods on the basis of deep learning to address the AMC problem in the field of communication. The classification methods are based on the end-to-end process, which performs feature extraction and classification in a unified framework, unlike the traditional methods. First, the communication signal dataset system was developed based on an actual geographical environment to provide the basis for related classification tasks. CNNs and LSTM were then used to solve the AMC problem. The models are capable of obtaining high-performing filters which significantly improve the capacity for feature representation for AMC. Furthermore, the modified classifiers based on the fusion model in serial and parallel modes are of great benefit to improving classification accuracy when the SNR is from 0 dB to 20 dB. The proposed methods in this paper achieve a much lower computational complexity during testing when compared with the training process. The serial fusion mode has the best performance compared with other modes. The probability matrices significantly reflect the shortcomings of the classifiers in this study. We will overcome these shortcomings with further research on AMC in the future [41,42].

## Figures and Tables

**Table 1:** Dataset descriptions.

Content	Detailed description
Modulation mode	Eleven types of single-carrier modulation modes (MASK, MFSK, MPSK, MQAM)
Carrier frequency	20 MHz to 2 GHz
Noise	0 dB to 20 dB
Attenuation	A fading channel based on a real geographical environment
Sample value	22,000 samples (11,000 training samples and 11,000 test samples)

**Table 2:** Training parameters and computational complexity of CNNs.

	Kernels	Parameters (M)	Training Time (s)	Testing Time (s)
CNN1 (with size 20)	8	1.537	72	0.4
	16	3.073	96	0.6
	32	6.146	118	1.1
CNN2 (with size 20)	8-8	1.539	96	1.0
	16-16	3.079	144	1.5
	32-32	6.166	250.5	2.85
CNN3 (with size 20)	8-8-8	1.540	148	1.55
	16-16-16	3.084	196	2.16
	32-32-32	6.187	420	4.3
CNN4 (with size 20)	8-8-8-8	1.541	165	2.3
	16-16-16-16	3.089	296.5	3.3
	32-32-32-32	6.207	507.5	5.9

**Table 3:** Classification accuracy of different methods without noise.

Methods	Wavelet/SVM	CNN	Bi-LSTM	Parallel Fusion	Serial Fusion
Accuracy	92.8%	91.2%	92.5%	93.1%	98.9%

**Table 4:** Classification accuracy of different methods with signal-to-noise ratio (SNR) from 0 to 20dB.

SNR Methods	20 dB	16 dB	12 dB	8 dB	4 dB	0 dB
Wavelet/SVM	85.2%	84.1%	83.2%	81.6%	79.0%	77.5%
CNN	86.1%	84.0%	82.1%	78.1%	73.6%	62.1%
Bi-LSTM	87.2%	84.9%	82.7%	77.5%	72.5%	66.0%
Parallel fusion	89.1%	85.2%	84.6%	80.0%	75.4%	67.9%
Serial fusion	98.2%	95.6%	94.3%	91.5%	86.2%	78.5%
